# Penile Epidermal Inclusion Cyst

**DOI:** 10.1155/2012/191343

**Published:** 2012-05-28

**Authors:** M. El-Shazly, A. Ghobashy, A. Allam, T. Alenezy, N. Alenezy, E. Yordanov, B. Hathout, R. Albunnai

**Affiliations:** Departments of Urology and Pathology, Farwaniya Hospital, Ardiya 92400, Kuwait

## Abstract

We report a case of epidermal inclusion cyst in a 32-year-old male. This was a complication of circumcision that was neglected over years to form stones and urethrocutaneous fistula. Complete excision of the cyst and repair of the fistula were performed successfully. Histopathological examination confirmed our diagnosis.

## 1. Introduction

Epidermal inclusion cyst is a rare complication of neonatal male circumcision. A search of all databases revealed that very few reports are available in the literature [1–6].

## 2. Case Report

Thirty-two-year-old patient presented to our department complaining of hard lesion on the ventral aspect of penile skin. The lesion has increased gradually in size over last 3 years. The patient reported passage of few drops of urine after micturition from 2 small opening close to the mass. He had a history of circumcision when he was 2 years old. Examination revealed subcoronal cyst with hard object inside like stones and 2 small urethrocutaneous fistulae. X-ray revealed radioopaque shadows suggesting stones.

**Figure 1:**
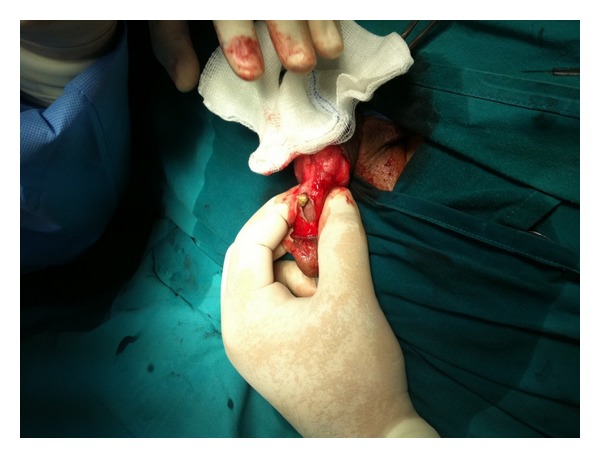
Retrieval of the stones from inside the cyst.

**Figure 2:**
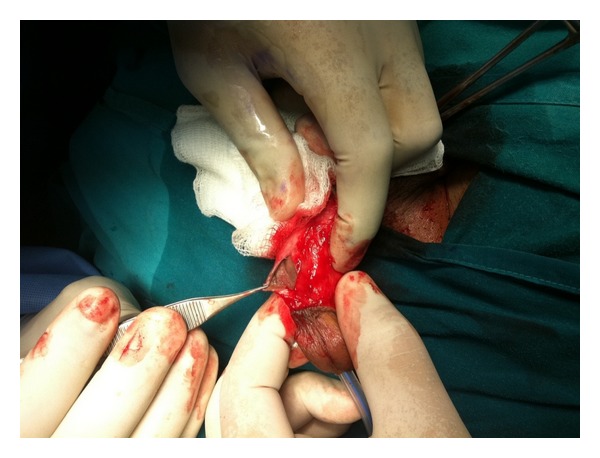
Inside of the cyst showing normal penile skin.

**Figure 3:**
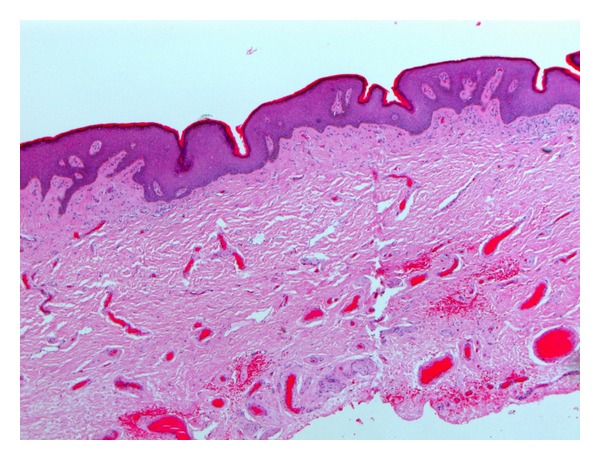
Histopathological picture confirms the diagnosis of penile epidermal inclusion cyst.

Subcoronal circumferential incision with degloving of penile skin was performed. The cyst was incised and revealed 4 different size stones (Figure 1). The inner side of the wall of the cyst proved to be penile skin suggesting the diagnosis of epidermal inclusion cyst (Figure 2). Complete excision of the cyst was done and the opening of the urethra was closed using continuous vicryl 6/0 sutures. A dartos flap was dissected from the ventral aspect of the penis and was sutured as a second layer over the urethra, then penile skin was closed. Catheter was removed after one week and patient had no recurrence of the urethrocutaneous fistula. The histopathological diagnosis was an epidermal inclusion cyst of the penis (Figure 3).

Epidermal inclusion cyst results from implantation and proliferation of epidermal element in the dermis [2]. Epidermal inclusion cyst can arise from surgical implantation of epidermal tissue, as in this patient, these cysts may also arise from the sequestration of epidermal rests during embryonic life, occlusion of the pilosebaceous unit, or traumatic implantation of epithelial elements [3].

Urethral diverticulum with stone formation is the most important differential diagnosis of this case. This was excluded after exploring the cyst and detection of penile skin as the wall of the cyst. In cases of urethral diverticulum, the wall should be urothelium not penile skin.

Epidermal inclusion cyst is a rare complication of male circumcision. It is the only report of penile epidermal inclusion cyst complicated with stones and urethrocutaneous fistula. Complete excision of the cyst and repair of fistula is easy and curative.

## References

[1] Muula AS, Prozesky HW, Mataya RH, Ikechebelu JI (2007). Prevalence of complications of male circumcision in Anglophone Africa: a systematic review. *BMC Urology*.

[2] Saini P, Mansoor MN, Jalali S, Sharma A (2010). Penile epidermal inclusion cyst. *Indian Journal of Pediatrics*.

[3] Okeke LI (2009). Epidermal inclusion cyst as a rare complication of neonatal male circumcision: a case report. *Journal of Medical Case Reports*.

[4] Wiswell TE, Geschke DW (1989). Risks from circumcision during the first month of life compared with those for uncircumcised boys. *Pediatrics*.

[5] Park HJ, Park NC, Park SW, Jern TK, Choi KU (2008). Penile epidermal inclusion cyst: a late complication of penile girth enhancement surgery. *Journal of Sexual Medicine*.

[6] Kaviani A, Hosseini J, Vazirnia AR (2009). A huge penile mass which turned out to be an epidermoid inclusion cyst. *Urology Journal*.

